# Structural insights into acetylated histone ligand recognition by the BDP1 bromodomain of *Plasmodium falciparum*

**DOI:** 10.1016/j.ijbiomac.2022.10.247

**Published:** 2022-10-31

**Authors:** Ajit Kumar Singh, Margaret Phillips, Saleh Alkrimi, Marco Tonelli, Samuel P. Boyson, Kiera L. Malone, Jay C. Nix, Karen C. Glass

**Affiliations:** a Department of Pharmacology, Larner College of Medicine, University of Vermont, Burlington, VT 05405, USA; b Department of Pharmaceutical Sciences, Albany College of Pharmacy and Health Sciences, Colchester, VT 05446, USA; c NMRFAM and Department of Biochemistry, University of Wisconsin-Madison, Madison, WI 53706, USA; d Molecular Biology Consortium, Advanced Light Source, Berkeley, CA 94720, USA

**Keywords:** *Plasmodium falciparum* (*P. falciparum*), Malaria, *P. falciparum* bromodomain-containing protein 1 (*Pf*BDP1), Bromodomain (BRD), Histones, Post-translational modifications (PTM), Chromatin reader domains, Epigenetics, X-ray crystallography, Nuclear Magnetic Resonance (NMR), Isothermal Titration Calorimetry (ITC)

## Abstract

*Plasmodium falciparum* requires a two-host system, moving between Anopheles mosquito and humans, to complete its life cycle. To overcome such dynamic growth conditions its histones undergo various post-translational modifications to regulate gene expression. The *P. falciparum* Bromodomain Protein 1 (*Pf*BDP1) has been shown to interact with acetylated lysine modifications on histone H3 to regulate the expression of invasion-related genes. Here, we investigated the ability of the *Pf*BDP1 bromodomain to interact with acetyllsyine modifications on additional core and variant histones. A crystal structure of the *Pf*BDP1 bromodomain (*Pf*BDP1-BRD) reveals it contains the conserved bromodomain fold, but our comparative analysis between the *Pf*BDP1-BRD and human bromodomain families indicates it has a unique binding mechanism. Solution NMR spectroscopy and ITC binding assays carried out with acetylated histone ligands demonstrate that it preferentially recognizes tetra-acetylated histone H4, and we detected weaker interactions with multi-acetylated H2A.Z in addition to the previously reported interactions with acetylated histone H3. Our findings indicate *Pf*BDP1 may play additional roles in the *P. falciparum* life cycle, and the distinctive features of its bromodomain binding pocket could be leveraged for the development of new therapeutic agents to help overcome the continuously evolving resistance of *P. falciparum* against currently available drugs.

## 1. Introduction

Eukaryotic cells accommodate nearly three meters of genetic information into a relatively small space within the nucleus by wrapping ~146 bp of double-stranded DNA around an octamer of histones to form the nucleosome [1–3]. In addition to this highly ordered packaging, the high affinity interactions between the histone proteins and DNA restricts the accessibility of the genetic information by various accessory proteins required to perform essential cell functions such as DNA replication, recombination, repair, and transcription [4–6]. The histone octamer is made up of the H3-H4 histone tetramer and two H2A-H2B histone dimers [2,3,7]. The protein sequence of histone H3 and H4 are highly conserved among all species ranging from yeast to humans, whereas the protein sequences of the H2A and H2B histones can vary greatly [8]. The histone proteins are highly basic in charge, and contain a globular C-terminal region along with a protease sensitive N-terminal tail that protrudes from the surface of the nucleosome core particle [9]. The flexible N-terminal tail region of the histone protein is abundant in lysine, arginine, and serine residues, which undergo various covalent post-translational modifications (PTMs) such as acetylation, phosphorylation, methylation, ubiquitination, and SUMOylation [6,9]. The histone tail PTMs are known to modulate the DNA-nucleosome interaction, and are also recognized by various accessory proteins required for gene activation and/or gene silencing [10]. Thus, the combinations of PTMs are also referred to as a histone code, which is an important epigenetic mechanism responsible for regulating access to the genetic information [5,9,11,12]. The enzymes responsible for managing the type and position of these specific PTMs, are known as writers (methylases, acetylases, kinases, and ubiquitinases), and erasers (demethylases, deacetylases, phosphatases) of the code [9,13]. There are also protein reader modules (bromodomains, chromodomains, WD40 repeat, and PHD fingers, etc.) that recognize the epigenetic signals provided by particular PTMs and recruit associated proteins/cellular machinery to genomic positions to carry out their function [14]. For example, bromodomains, which are known to recognize acetylated lysine residues on the N-terminal histone tails, share an evolutionarily conserved structural fold comprised of four alpha-helices connected by two variable-length loops, which form the acetyllysine binding pocket [15]. In humans, there are approximately 60 human bromodomains divided into eight subfamilies, and they are often found in chromatin remodeling proteins and transcription factors that are involved in essential cellular functions including transcription, DNA replication, and DNA repair [15].

**Fig. 1.**
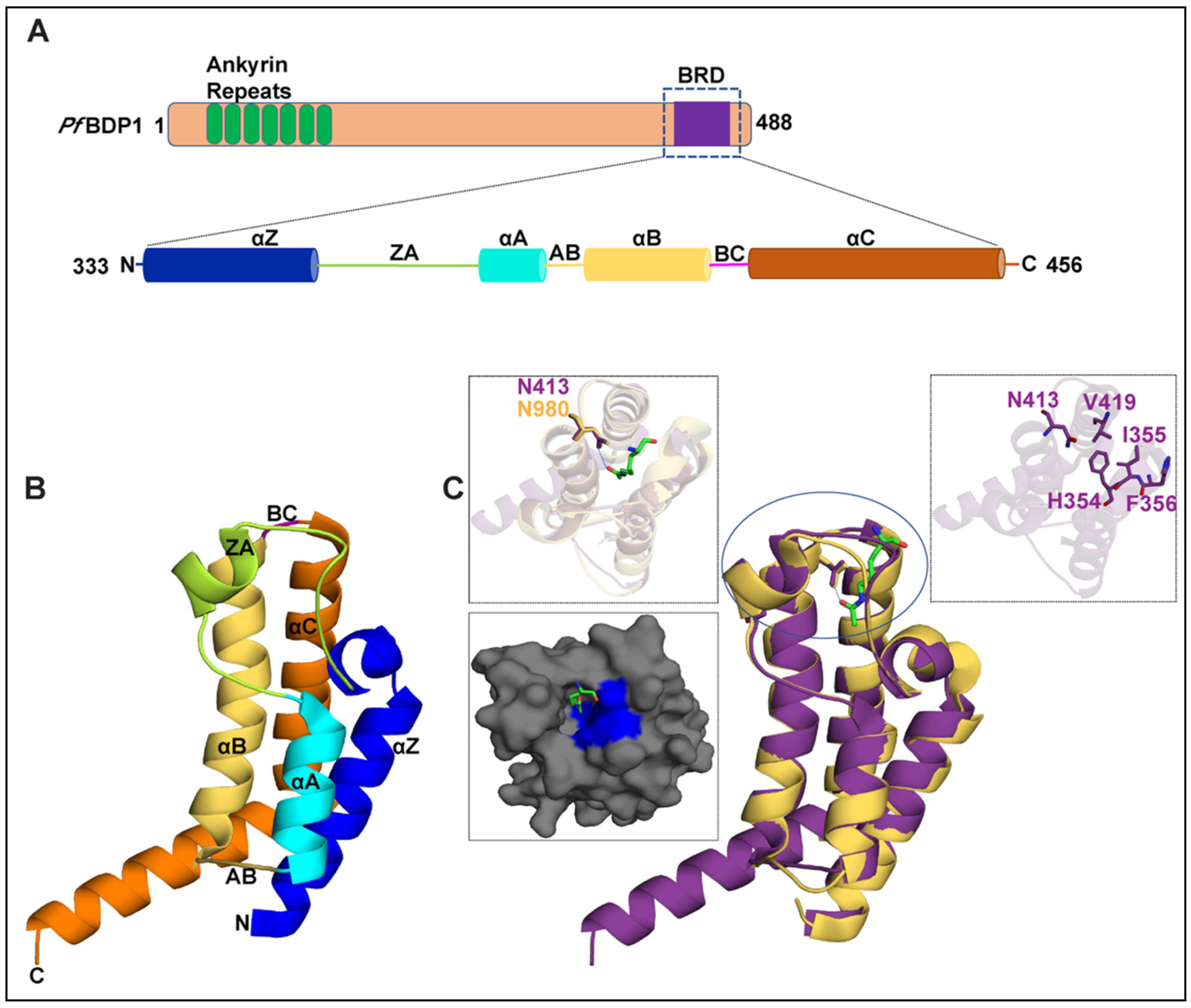
Structural features of the *Pf*BDP1 protein. (A) Domain organization of *Pf*BDP1 protein, where the ankyrin repeats are highlighted in green, loop regions in tan, and the bromodomain (BRD) in purple. The BRD is further expanded to highlight the secondary structure elements assigned to regions of the amino acid sequence based on the 2.0 Å crystal structure of the *Pf*BDP1-BRD (333–456) (PDB ID: 7M97). (B) Cartoon representation of the *Pf*BDP1-BRD (333–456) (PDB ID: 7M97) structure, where the αZ helix is blue, the αA helix is cyan, αB helix is yellow, αC helix is orange, the ZA loop is green, and the BC loop is magenta. (C) Structural alignment of *Pf*BDP1-BRD (333–456) in purple with the TIF1α bromodomain (PDBID: 3O35) in yellow. The acetyllysine found in the TIF1α bromodomain is colored green and represented as a stick model. The left panel insert shows a zoomed in view of the binding pockets and coordination of acetyllysine by the TIF1α bromodomain. The surface representation of the predicted binding pocket of *Pf*BDP1-BRD (333–456) is shown below where the hydrophobic residues are highlighted in blue and the acetyllysine group from the overlaid TIF1α bromodomain structure is in green. The right panel insert shows the *Pf*BDP1-BRD structure in purple with the conserved asparagine N413, the gatekeeper residue V419, and the HIF motif (354–356 aa) residues depicted as sticks.

Bromodomain-containing proteins have also been identified in parasitic organisms including *Plasmodium falciparum* [16]. This unicellular eukaryote exhibits a very complex life cycle for its survival and replication. It requires a two-host system, moving between the Anopheles mosquito (invertebrate) and humans (vertebrate), and invades multiple cell types such as insect cells, hepatocytes, and red blood cells [17]. To adapt to such dynamic environmental conditions its genome undergoes various post-translational modifications to regulate the specific activation or inactivation of genes at particular stages [18]. The genome of the *Plasmodium falciparum* is organized by the four canonical core histones (H3, H4, H2A, and H2B), as well as variant histones H2A.Z, H2B.Z, H3.3, and CenH3 [19–21]. These histones are reported to be enriched with acetylated lysine residues in the transcriptionally active euchromatin region. Using mass spectrometry techniques, several acetylated lysine residues have been identified on the N-terminal tail region of *P. falciparum* histone proteins including H3 (K4ac, K9ac, K14ac, K18ac), H4 (K5ac, K8ac, K12ac, K16ac, K20ac), H2A.Z (K11ac, K15ac, K19ac), and H2B.Z (K3ac, K8ac, K13ac, K14ac, K18ac) [19,20,22,23]. Some of these modifications are known to occur at specific stages of its life cycle, namely histone H3K9ac/H3K14ac, and their function has been linked to the activation of invasion-related genes required for entry into red blood cells [24]. The *P. falciparum* genome encodes histone acetyltransferases (HATs), histone deacetylases (HDACs), and histone methyltransferases (HMTs) that dynamically regulate the presence of specific PTMs [25,26]. The *P. falciparum* genome also encodes for ten bromodomain-containing proteins namely, Bromodomain Proteins 1–8 (*Pf*BDP1, *Pf*BDP2, *Pf*BDP3, *Pf*BDP4, *Pf*BDP5, *Pf*BDP6, *Pf*BDP7, *Pf*BDP8, GCN5 (histone acetyltransferase GCN5), and SET1 (SET domain protein 1)), which likely recognize acetylated lysine modifications. Recent studies on *Pf*BDP1 have shown that it is a multidomain protein with a predicted ankyrin repeat in the N-terminal region, and a bromodomain (BRD) near the C-terminus (Fig. 1A) [16,24,27–29]. *Pf*BDP1 is thought to play an important role in the activation of invasion-related genes in the asexual stage as its knockdown drastically affects the replication of *P. falciparum* in red blood cells. The bromodomain of *Pf*BDP1 was shown to recognize specific acetylated lysine marks, such as histone H3K9ac and H3K14ac, over methylated or unmodified histone H3 [24]. In addition, histone peptide pull-down assays also indicate that *Pf*BDP1 can interact with acetyllysine modifications on histone H4, and the histone variants H2B.Z and H2A.Z. [22]. Interestingly, *Pf*BDP1 appears to form a core complex with two other bromodomain-containing proteins, *Pf*BDP2 and *Pf*BDP7, which were also enriched with acetylated histones in the pull-down assays [22,24,27]. *Pf*BDP7 is an essential gene in *P. falciparum* that was recently shown to co-localize with the genome-wide binding sites of *Pf*BDP1. Both proteins are commonly found at the promoters of invasion genes, however, *Pf*BDP1 and *Pf*BDP7 are also enriched in heterochromatin at genes encoding the variant surface antigens, which contribute to parasite infection. Thus, they are also thought to function as an important regulatory complex for silencing the expression of variant surface antigen [27]. Similarly to the Spt/Ada/Gcn5 acetyltransferase (SAGA), and the remodel the structure of chromatin (RSC) chromatin remodeling complexes, which contain multiple bromodomain proteins, *Pf*BDP7 has been shown to interact with *Pf*BDP1 and *Pf*BDP2, likely allowing the complex to recognize a specific subset of acetylation marks on histone proteins [22,24,27].

Among the five *Plasmodium* species, *Plasmodium falciparum* is the largest contributor to malaria infections in humans. According to the 2019 WHO report, nearly half of the world’s population is at risk of malaria, which includes around 229 million active cases and 409,000 deaths annually. The most affected groups from malaria includes children below the age of five. While the development of a vaccine to prevent this deadly disease shows great promise, human mortality is still predicted to increase [30,31]. This is due in part to low vaccination rates in young children, and also due to evolutionary processes that drive parasite resistance against the currently available antimalarial drugs [32]. Thus, new therapeutics will continually be needed to treat infected patients. A better understanding of the *Plasmodium falciparum* life cycle, and the molecular mechanisms driving infection and disease progression are needed to develop additional anti-malarial strategies. Bromodomains are validated drug targets for the treatment of various diseases [33]. Additional structural and functional studies on the *Pf*BDP1 bromodomain will provide valuable information in search of small molecule inhibitors against it.

We hypothesized that the *Pf*BDP1-BRD would recognize multiple acetylation modifications, similarly to many of the human BRD counterparts. As such, we examined the structural fold of the *Pf*BDP1-BRD and carried out a systematic investigation of the bromodomain binding activity with acetylated histone ligands. We used X-ray crystallography to structurally characterize the *Pf*BDP1-BRD, and *in vitro* Isothermal Titration Calorimetry (ITC) and Nuclear Magnetic Resonance (NMR) binding studies, to investigate its ability to recognize specific acetyllysine modifications on histones H3, H4, H2A.Z, and H2B.Z. Our results outline the structural differences between the *Pf*BDP1 bromodomain compared to human bromodomains, and we characterized its functional activity by identifying its preferred histone ligands. Our results also provide new insights into the potential role(s) of *Pf*BDP1 in the life cycle of *Plasmodium falciparum*. Together, our structure-function approach will aid in the design of specific therapeutics to inhibit the activity of *Pf*BDP1, which may offer a mechanism to restrict the replication of *P. falciparum* in red blood cells to treat the disease.

## 2. Results

### 2.1. Crystal structure of the PfBDP1-BRD

Bromodomains are conserved reader domains that are known to recognize acetyllysine marks on the N-terminal tails of histone proteins. Prior research on the *Pf*BDP1-BRD indicates that it plays an important role in recognizing acetylated histone H3 to regulate the expression of invasion-related genes, which facilitates parasite entry into red blood cells. The *Pf*BDP1 protein is organized into multiple domains consisting of seven ankyrin repeats at the N-terminus and a bromodomain towards the C-terminal region (residues 333–456) (Fig. 1A). However, there is no information available on the molecular mechanisms utilized by the *Pf*BDP1-BRD to coordinate histone ligand recognition and binding. To structurally characterize the *Pf*BDP1-BRD in complex with the histone H3K14ac ligand, an expression construct containing residues 333–456 was used for the crystallization trials. Protein crystals of *Pf*BDP1-BRD (333–456) grown in complex with the H3K14ac ligand diffracted to 2.0 Å. The structure was solved by molecular replacement with a monomer of the *Pf*BDP1 bromodomain in the asymmetric unit. The structure was refined to a final Rwork and Rfree values of 19.49% and 23.65%, respectively, and the data collection and refinement statistics are shown in Table 1. Disappointingly, no electron density was observed for the histone H3K14ac ligand. However, the final structure of *Pf*BDP1-BRD (333–456) protein possesses the conserved bromodomain fold consisting of four left-handed alpha helices (Fig. 1B). The four-helix bundle is arranged in two halves connected by the AB and BC loops (residues 389–393 and 413–417). The first half is comprised of the αZ (333–357 aa) and αA (379–389 aa) helices. The end of the αZ helix encodes a ‘HIF’ shelf that forms the bottom of the binding pocket, and the long and variable ZA loop (358–378 aa) frames one side of the binding pocket before connecting to the αA helix. Part of the ZA loop also forms a short α-helix from residues 369–374, which is a conserved structural feature of the bromodomain binding pocket [15]. The second half of the conserved bromodomain fold is comprised of the αB helix (394–413 aa) and the αC helix (417–455 aa) that form the other side of the binding pocket. The αB helix is 19 aa shorter than the αC helix and is connected by a 3-residue long BC loop, which contains the conserved asparagine residue N413 that is involved in the coordination of the acetyllysine moiety (Fig. 1C). Thus, the *Pf*BDP1 bromodomain possesses the characteristic structural features that enable the recognition and binding of acetyllysine post-translational histone modifications.

### 2.2. Comparison of the structural features of the PfBRD1-BRD binding pocket with the human TIF1α-BRD/TRIM24 PHD-BRD

Bromodomains are known to specifically recognize acetylated lysine modifications through their conserved asparagine and gatekeeper residues. To identify these residues in *Pf*BDP1-BRD its structure was compared with the human bromodomains in a complex with an acetylated histone ligand. *Pf*BDP1-BRD shows the closest structural similarity with the TIF1α-BRD (TRIM24 PHD bromodomain) acetyllysine complex (PDB ID: 3O35) with an RMSD (root mean square deviation) value of 0.88 Å. This structure was used for depicting the *Pf*BDP1-BRD acetyllysine binding pocket. Like other bromodomains, the predicted acetyllysine binding pocket of *Pf*BDP1-BRD (333–456) has a conserved asparagine residue (Asn413) responsible for making a hydrogen bond to the acetyllysine, while the gatekeeper residue valine (Val419) and the HIF shelf motif (res 354–356) are important for ligand binding specificity (Fig. 1C). Similar to human bromodomains, it also has an abundance of hydrophobic residues, where six hydrophobic residues are present in the αZ, αB, αC helices, and 4 hydrophobic residues are present in the ZA loop (Fig. 1C). In conclusion, our structural analysis indicates *Pf*BDP1-BRD can also specifically recognize acetyllysine marks on histone proteins.

### 2.3. Comparison of PfBDP1-BRD with human bromodomains

To further define the unique features of the *Pf*BDP1-BRD, we carried out an in-depth structural analysis to compare the similarities and differences of the *Pf*BDP1-BRD with the structures of human bromodomains. In humans, there are 61 bromodomains that are classified into eight families based on a structural alignment [15]. We compared our structure of the *Pf*BDP1-BRD to one member from each family that was selected based on the highest sequence identity and the availability of a deposited crystal structure in the PDB. Thus, we used the structure of the CECR2-BRD from family I, the second BRD from BRD4 in family II, the CREBBP-BRD in family III, the ATAD2B-BRD in family IV, the TIF1α-BRD from family V, the TRIM28-BRD in family VI, the second BRD from TAF1 in family VII, and the third BRD from PB1 in family VIII (3). Interestingly, the *Pf*BDP1-BRD has the highest sequence identity (48.44%) with the family III BRD of CREBBP, while it had the lowest sequence identity with the family VI BRD of TRIM28 (22.95%) (Table 2 and Suppl. Fig. 2A).

To investigate the structural similarities and differences of *Pf*BDP1-BRD with each of these human bromodomain proteins, we first calculated the RMSD using the PDBeFold online server with our *Pf*BDP1-BRD crystal structure as the query structure [34]. The structural fold of *Pf*BDP1-BRD shows the highest similarity with the BRD of CECR2 in family I, having an RMSD value of only 0.88 Å. In contrast, the largest structural differences were observed between the *Pf*BDP1-BRD and the family IV BRD of ATAD2B, which displayed an RMSD value of 2.13 Å (Table 2 and Suppl. Fig. 2B).

**Fig. 2.**
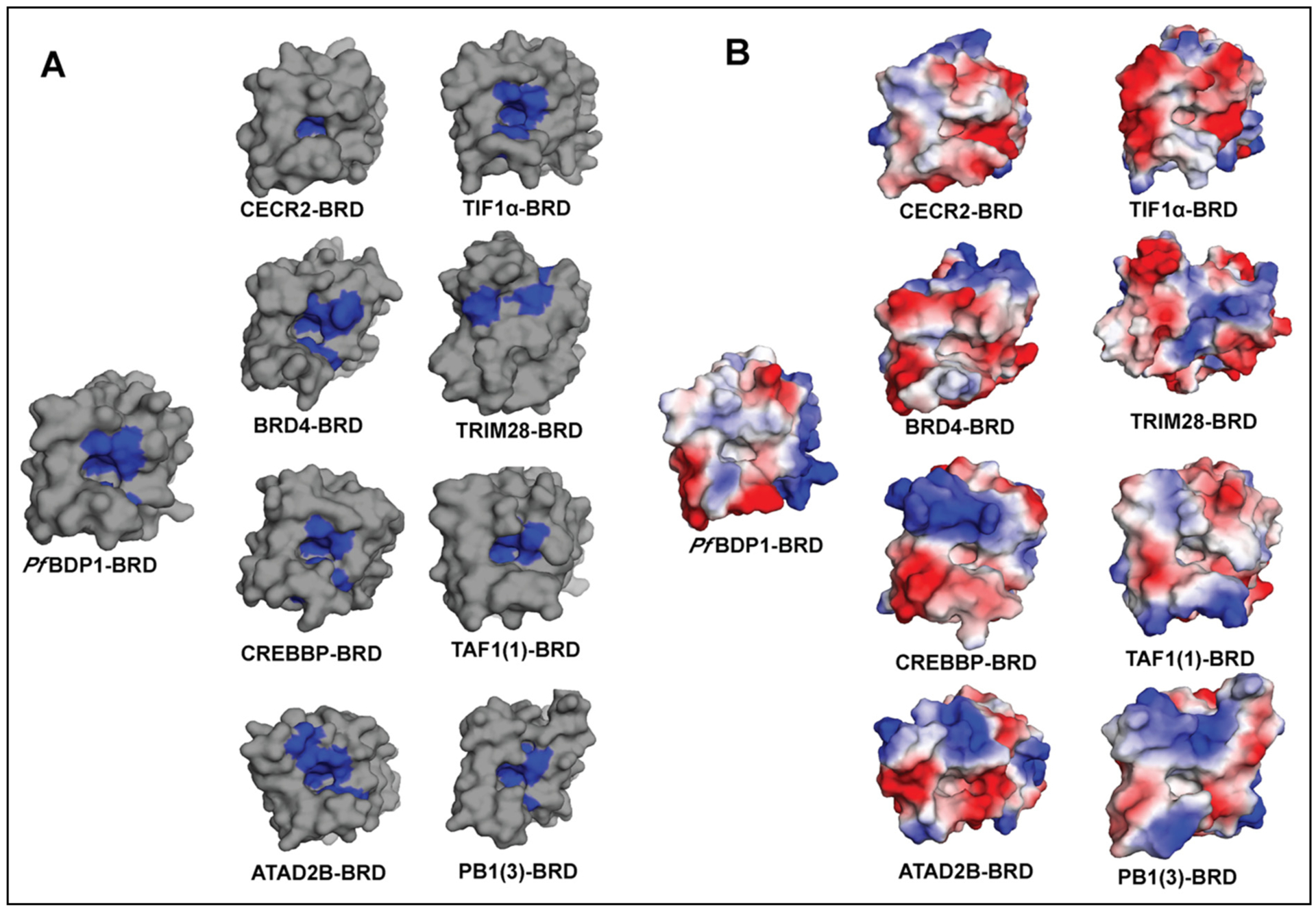
Comparison of hydrophobic and electrostatic features of the BRD binding pockets. (A) Surface representation of the *Pf*BDP1-BRD (331–456) is depicted alongside representative human bromodomains from families I–VIII. The blue regions in the left panel highlight the hydrophobic residues near the acetyllysine binding pocket, while the gray region shows the overall surface of the bromodomains. (B) An electrostatic surface potential map was generated using PyMOL [47] for the *Pf*BDP1-BRD (331–456) and representative human bromodomains. Electrostatic surface potentials are shown over a range of 88.229kT/e (red) and +88.229kT/e (blue).

The crystal structure of *Pf*BDP1-BRD was also compared with the human bromodomains to understand the differences in amino acid composition in the acetyllysine binding pocket with a particular focus on hydrophobic and charged amino acid residues, which are known to play an important role in the recognition of acetylated lysine residues. As shown in Fig. 2A and Table 2 the *Pf*BDP1-BRD (PDB ID: 7M97) has 10 hydrophobic residues in the acetyllysine binding pocket, which is similar to the number of hydrophobic residues found in the CREBBP-BRD. Whereas it has fewer hydrophobic residues than bromodomains of BRD4, KIAA1240, TIF1α, TAF1, and PB1(3), and more than the CECR2 and TRIM28 human bromodomains. The electrostatic surface potential map (Fig. 2B) shows the *Pf*BDP1-BRD is most similar to the ATAD2B and CECR2 bromodomains with 6 charged amino acid residues (acidic and basic) in the acetyllysine binding pocket. Its binding pocket appears to be more highly charged than the BRDs of BRD4, CREBBP, TIF1α, and TAF1, and less charged in comparison to the TRIM28 and PB1(3) bromodomains (Table 2 and Fig. 2B). Our systematic analysis of the BRD binding pockets illustrates that besides having a close structural similarity with human bromodomains, *Pf*BDP1-BRD has a unique chemical composition in the acetyllysine binding pocket, which may help in designing a specific drug inhibitor against *Pf*BDP1-BRD.

### 2.4. The PfBDP1-BRD preferentially interacts with acetylated histone H4 ligands

**Fig. 3.**
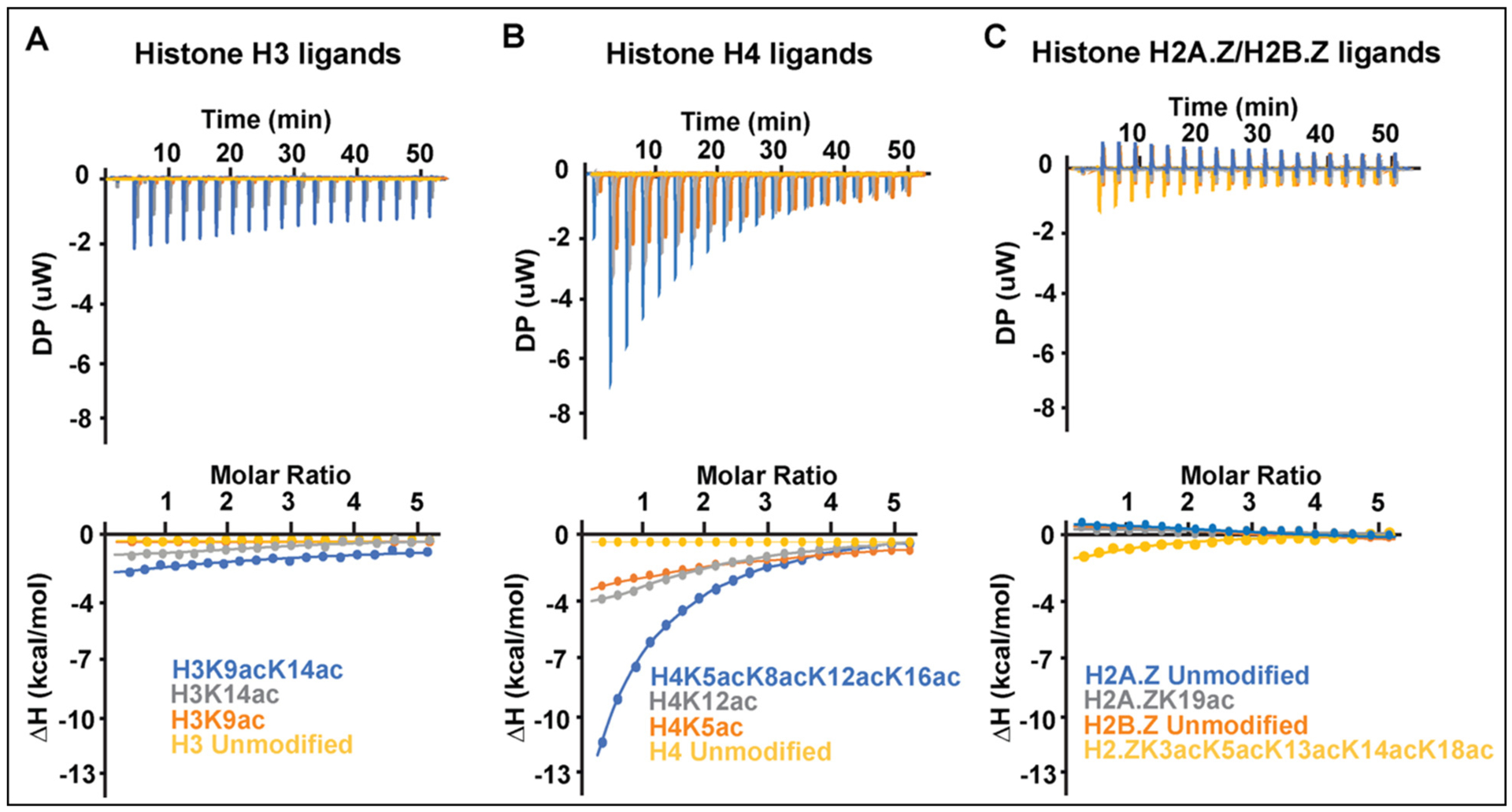
Superimposed ITC enthalpy plots for the binding of *Pf*BDP1-BRD with acetylated histone ligands. (A) *Pf*BDP1-BRD with histone H3 peptide ligands, where unmodified H3 is shown in yellow, H3K9ac in orange, H3K14ac in gray and H3K9acK14ac in blue. (B) *Pf*BDP1-BRD with histone H4 peptide ligands, where the unmodified H4 is shown in yellow, H4K5ac in orange, H4K12ac in gray, and H4K5acK8acK12acK16ac in blue. (C) *Pf*BDP1-BRD with the histone H2A.Z and H2B.Z peptide ligands, where unmodified H2A.Z is shown in blue, H2A.ZK19ac in gray, H2B.Z unmodified in orange, and H2B.ZK3acK8acK13acK14acK18ac in yellow.

Bromodomains are known to play a major role in decoding the epigenetic information by recognizing acetyllysine modifications to recruit bromodomain-containing proteins and any associated proteins/complexes to the chromatin [35]. The *Pf*BDP1-BRD was previously shown to interact with histone H3 acetylated at lysine 9 and 14 (H3K9ac and H3K14ac), and this activity was required for the activation of invasion related genes in *Plasmodium falciparum* [24]. However, characterization of the specific binding interactions of the *Pf*BDP1-BRD with PTM histone ligands was only carried out with a limited number of unmodified, acetylated, and methylated lysine residues on histone H3 [24]. Human bromodomains are frequently able to recognize multiple acetylation modifications on the core histones. For example, the well-studied BET bromodomain family binds to single and multiple acetyllysine mark combinations on histones H3 and H4 [15,35,36]. Based on the observed structural similarities between the *Pf*BDP1-BRD and human bromodomains as described above, we hypothesized that it would likely interact with additional acetyllysine modifications on both histone H3 and H4. To investigate this, we carried out isothermal titration calorimetry (ITC) binding assays with a library of *P. falciparum* histone peptides that contain acetyllysine modifications at all possible positions within the N-terminal tail of each histone. As shown in Table 3 we confirmed the interaction of *Pf*BDP1-BRD with the di-acetylated H3K9acK14ac (K_D_ = 1125 ± 181 μM), and mono-acetylated H3K14ac (K_D_ = 1215.0 ± 162.3 μM) histone ligands by ITC. However, the isothermal enthalpy plots showed no binding to the H3K9ac or the unmodified histone H3 ligands (Table 3, Fig. 3B, Suppl. Fig. 3). On the other hand, when the binding activity of *Pf*BDP1-BRD was tested against histone H4 peptides mono-acetylated at lysine residue K5, K8, K12, K16, and K20, it was able to recognize the H4K5ac (K_D_ = 1210 ± 208 μM) and H4K12ac (K_D_ = 1070 ± 158 μM) ligands, but did not interact with H4K8ac, H4K16ac, H4K20ac, or the unmodified H4 ligands. Interestingly, the *Pf*BDP1-BRD is able to select for di-acetylated H4 ligands including H4K5acK8ac (K_D_ = 1510.0 ± 168.0 μM) and H4K5acK12ac (K_D_ = 621.0 ± 28.3 μM), but it demonstrated the strongest binding affinity with the tetra-acetylated histone H4 peptide (H4K5acK8acK12-acK16ac, K_D_ = 117.0 ± 11.3 μM) (Table 3, Fig. 3B, Suppl. Fig. 3).

A recent study by Hoeijmakers, et al., used histone peptide pull-down coupled to mass spectrometry to identify histone readers and associated proteins in nuclear extracts from blood-stage *P. falciparum* parasite cultures [22]. They discovered that the *Pf*BDP1-BRD was enriched at acetyllysine modifications on the parasite-specific tails of histone variants H2A.Z and H2B.Z [22]. Thus, we carried out additional ITC binding assays to further evaluate the interaction of *Pf*BDP1-BRD with histone H2A.Z containing acetylated lysine at residues K11, K15, and K19, and with histone H2B.Z acetylated at positions K3, K8, K13, K14, and K18. However, in our study, we did not observe any interactions of the *Pf*BDP1-BRD with the mono-acetylated histone variants H2A.Z and H2B.Z. To further examine if multiple acetyllysine modifications may be required to recruit the *Pf*BDP1-BRD to histone variants we also tested its ability to bind the penta-acetylated H2B.ZK3acK8acK13acK14acK18ac, and it does show a weak interaction with this ligand (Table 3, Fig. 3B, Suppl. Fig. 3). Taken together, our results indicate that the *Pf*BDP1-BRD recognizes specific acetylated lysine marks found on histones H3 and H4, and preferentially binds to histone H4 when it contains multiple acetyllysine residues. This suggests that effector-mediated recognition of the histone tails by *Pf*BDP1-BRD is dependent on the combinatorial readout of multiple post-translational modifications over a span of amino acids on the histone tails.

**Fig. 4.**
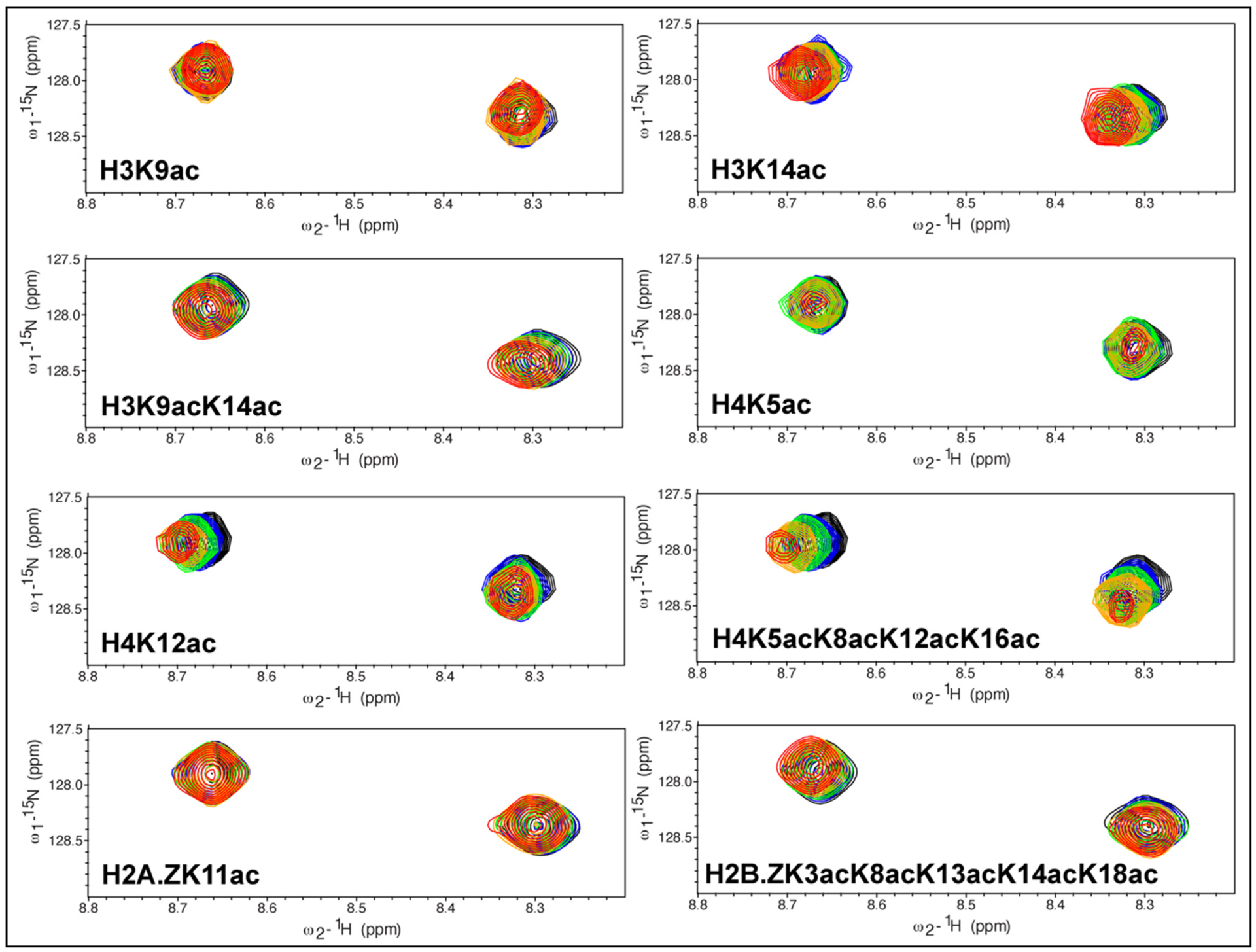
Interaction of the *Pf*BDP1-BRD with acetylated histone ligands. Superimposed 2D ^15^N-^1^H HSQC spectra of the ^15^N-labeled *Pf*BDP1-BRD collected in NMR titration experiments with the indicated histone peptides. The peaks of the *Pf*BDP1-BRD apo protein are shown in black. The titration of *Pf*BDP1-BRD with histone peptides was performed at increasing concentrations of each ligand and include 1:0.5 M ratio (blue), 1:1 M ratio (green), 1:2.5 M ratio (orange) and 1:5 M ratio (red). Each spectrum is labeled with the histone peptide used in the titration.

To further confirm the binding of acetylated histone peptides to the *Pf*BDP1-BRD we used NMR to carry out chemical shift perturbation studies (CSPs). We recorded two-dimensional ^15^N-^1^H heteronuclear single quantum coherence (HSQC) spectra and examined the changes in chemical shift of apo-bromodomain backbone amide peaks upon addition of unmodified and acetylated histone H4, H3, H2A.Z, and H2B.Z peptides. The absence of CSPs upon the addition of unmodified histone peptides to the *Pf*BDP1-BRD indicates no interaction (Suppl. Fig. 4), whereas distinct CSPs observed for the *Pf*BDP1-BRD residues in the presence of acetylated histone peptides confirm the binding interactions observed by ITC (Fig. 4). Among the acetylated histone H4 peptides (residues 1–24) tested, the tetra acetylated H4K5acK8acK12acK16ac showed the most significant CSPs. This is supported by our ITC data, which shows binding of K_D_ = 173.0 ± 11.3 μM, and is higher than the binding affinities for H4K5ac (K_D_ = 1210 ± 208 μM) and H4K12ac (K_D_ = 1070 ± 158 μM). Similar to the unmodified histone H4, there were no CSPs observed upon the addition of the unmodified histone H3 peptide (Suppl. Fig. 4). Interestingly, although the binding affinity of H3K9acK14ac was stronger than the H3K14ac ligand, we did not observe any CSPs in the presence of the singly modified histone H3K9ac ligand. This is complementary to our ITC data which also demonstrates no binding for the H3K9ac peptide. Addition of the H3K14ac histone peptide induced only weak CSPs. We also confirmed no binding to the H2A.ZK11ac ligand as illustrated by the absence of CSPs. However, we did observe weak binding of the H2B.ZK3acK8acK13acK14acK18ac histone ligand as illustrated by the presence of CSPs in the NMR spectra. In conclusion, our NMR titration experiments supplement our ITC data and confirm that the histone H4K5acK8acK12acK16ac peptide binds with the highest affinity to *Pf*BDP1-BRD, followed by the H4K12ac, H3K14ac, and the H2B.ZK3acK8acK13acK14acK18ac histone peptides.

## 3. Discussion

In this study, we report the highest resolution crystal structure for the *Pf*BDP1-BRD currently deposited in the PDB, and confirm it contains the conserved bromodomain fold and a functional acetyllysine binding pocket. Our ITC and NMR binding studies of *Pf*BDP1-BRD with acetylated histone ligands indicate it can specifically recognize the acetyllysine modifications on histones H3, H4, and H2B.Z. We demonstrated that the *Pf*BDP1-BRD binds to mono- and di-acetylated histone H3K14ac and H3K9acK14ac, respectively. These results are similar to what was previously reported by Josling et al. [24], however, we did not observe any interaction with the mono-acetylated histone H3K9ac ligand. Furthermore, our binding studies identified additional interactions of the *Pf*BDP1-BRD with histone H4 ligands that are mono-acetylated (H4K5ac, H4K12ac, H4K20ac), di-acetylated (H4K5acK8ac, H4K5acK12ac), and tetra-acetylated (H4K5acK8acK12acK16ac). Mass spectrometry experiments conducted by Hoeijmakers et al. suggested that *Pf*BDP1 interacts with acetylation marks on histone H2B.Z [22]. Our binding studies indicate that the *Pf*BDP1-BRD does not interact with monoacetylated H2A.Z and H2B.Z ligands, but the ITC and NMR titration assays support a weak interaction that may exist with the pentaacetylated histone H2B.ZK3acK8acK13acK14acK18ac ligand. To date *Pf*BDP4-BRD in complex with an acetylated histone peptide (PDBID 5VS7) is the only ligand bound crystal structure available for *P. falciparum* bromodomains. From our interaction studies, we demonstrated that the *Pf*BDP1-BRD recognizes the tetra-acetylated histone H4 peptide with the highest affinity. Since humans are one of the primary hosts for *Plasmodium falciparum*, which in turn causes malaria, we carried out a detailed analysis comparing the *Pf*BDP1-BRD with 61 bromodomains identified in humans [15]. *Pf*BDP1-BRD shows high structural identity with the human bromodomains, indicating it likely performs a similar function to its human counterparts. We found that although the *Pf*BDP1-BRD can be structurally aligned with the human bromodomains with an RMSD of <2.1 Å, it has a low sequence identity (>50%) with these proteins. However, the significant differences in primary sequence between *Pf*BDP1-BRD and human bromodomains, suggest that the *Pf*BDP1-BRD utilizes a unique molecular mechanism to select for specific acetyllysine modifications on the *P. falciparum* histones. For example, *Pf*BDP1-BRD has the highest structural identity with the TIF1α-BRD (RMSD = 0.88 Å), but only 39.58% sequence identity. Ligand binding studies on the TIF1α-BRD show that it binds to the acetylated histone peptide H4K16ac with a K_D_ of 92.6 ± 1.9 μM by ITC, whereas the *Pf*BDP1-BRD does not show any interaction with the H4K16ac mark [15]. In addition, the *Pf*BDP1-BRD has low structural (RMSD = 2.0 Å) and sequence identity (38.10%) with the TAF1-BRD(1). As expected the TAF1-BRD(1) does not have any overlapping binding activity with the *Pf*BDP1-BRD [15]. Differences in the primary sequence between the *Pf*BDP1-BRD and human bromodomains contribute to the unique features of the acetyllysine binding pocket. Our structural analysis revealed that the number of hydrophobic residues and surface electrostatics of the *Pf*BDP1-BRD has some similarities with the CREBBP and TRIM28 human bromodomains, but it contains an exclusive combination of amino acids that determine ligand specificity. For example, the conserved acetyllysine anchor residue (Asn413) in the *Pf*BDP1-BRD is close to the gatekeeper residue at the top of the αC helix. The small hydrophobic Val419 gatekeeper residue in *Pf*BDP1-BRD is conserved with the family II BET-BRDs, which also have the ability to select for multiply acetylated histones [15]. However, instead of the WPF shelf residues found in the family II BET-BRDs, the *Pf*BDP1-BRD has a HIF shelf that is only observed in the human family IV BRPF3-BRD. Thus, the *Pf*BDP1-BRD appears to be a cross between two human bromodomain families by having the ‘wide’ acetyllysine binding pocket observed in the BET bromodomains, combined with the shelf motif from BRPF3, which has a ‘keyhole’ type pocket due to its large Phe gatekeeper residue [15,37]. This distinctive arrangement of specificity determinants in the binding pocket, in combination with a unique set of hydrophobic and electrostatic features will be important drivers underlying the molecular mechanism of ligand recognition by the *Pf*BDP1-BRD. It also provides a valuable opportunity to design small molecule inhibitors that can selectively target the *Pf*BDP1-BRD via the rational design of chemical-based interactions optimized to take advantage of these features.

Bromodomain-containing proteins are known to play a major role in the regulation of gene activation or inactivation and have been validated as a potent therapeutic target for the treatment of various diseases such as cancer, neurological disorders, and inflammation [33]. Currently, there are several small-molecule bromodomain inhibitors in clinical trials against cancer, and some BRD inhibitors may also be useful for treating neurological disorders due to their ability to cross the highly selective blood-brain barrier [38]. To date there are not any effective small molecule inhibitors available that target the *Pf*BDP1-BRD to treat malaria. Thus, the new data obtained in this study enables a further understanding of the structure and function of *Pf*BDP-BRD and will be a valuable resource in the search for small molecule inhibitors using a similar approach employed to target human bromodomains.

The *Pf*BDP1 protein plays an essential role in the *P. falciparum* life cycle and survival, supporting the idea that this protein could be an effective anti-malarial drug target [16,24,29]. With resistance quickly developing against antimalarial drugs, it has become increasingly important to understand the etiology and molecular mechanisms driving malaria infections in order to identify additional drug targets for the development of new anti-malarials. An approach using a combination of anti-malarial drugs that target multiple life cycle stages of *P. falciparum* would likely increase the efficacy of treatment while also reducing the development of resistance. The identification of target proteins that have a significant role in various stages of life cycle of *P. falciparum* may provide additional therapeutic utility. Histone post-translational modifications are known to change dynamically during different stages of the cell cycle, throughout development, and as disease progresses [39]. Thus, the interaction of the *Pf*BDP1-BRD with particular histone modifications may be important for regulating specific stages of the *P. falciparum* life-cycle. Our study establishes an additional activity of the *Pf*BDP1-BRD in recognizing histone H4 and H2B.Z acetylation marks, whose functional significance with respect to the regulation of gene expression in *P. falciparum* has yet to be explored. The *Pf*BDP1 protein has also been shown to form a multi-subunit complex with *Pf*BDP2 and/or *Pf*BDP7 [24,27,40]. In addition to the C-terminal bromodomain, the full-length *Pf*BDP1 protein contains 7 ankyrin repeats located near the N-terminus, which may function as a protein-protein interaction domain. Thus, the interaction of the bromodomain with acetylated H3, H4, and H2B.Z histones could recruit *Pf*BDP1 to chromatin, and the ankyrin repeats may serve as a bridge between *Pf*BDP1 and *Pf*BDP2/*Pf*BDP7. This would allow them to work together in a complex to regulate gene activation and inactivation processes. Our studies suggest that *Pf*BDP1 likely performs important chromatin regulatory functions at various stages of the *P. falciparum* life cycle and provide additional support for its utility as a target in the development of new approaches for anti-malarial therapeutics.

Although ten bromodomain-containing proteins have been identified in *P. falciparum*, functional studies are available on only three of these proteins including, *Pf*BDP1, *Pf*GCN5, and *Pf*BDP7 [24,27,41]. The domain organization illustrates that all of them except *Pf*BDP3 have a C-terminal bromodomain, which is located on the N-terminus, while *Pf*BDP4 has two consecutive bromodomains in the C-terminal region [42]. A phylogenetic analysis of bromodomains in Protozoan parasites indicates that *Pf*BDP1, *Pf*BDP2, and *Pf*BDP3 cluster into an evolutionarily related family, which suggests that *Pf*BDP2/3 could perform functions similar to *Pf*BDP1 [16]. Furthermore, *Pf*BDP1 is evolutionary and most closely related to the *Toxoplasma gondii* Bromodomain Protein 1 (*Tg*BDP1) [16]. As a functional ortholog, *Tg*BDP1 may have similar biological roles in the development of disease. Thus, our current study outlining the structure and function of *Pf*BDP1-BRD may also translate to the *Tg*BDP1-BRD and assist in the design of bromodomain inhibitors that specifically target both *T. gondii* and *P. falciparum*. *Pf*BDP1 is a multidomain protein, and in this study only the C-terminal BRD region was tested for binding, therefore there are some caveats to this study. It is possible that the other domains contribute to histone ligand recognition in physiological contexts. Also, while measuring the binding affinities for chromatin reader domains with specifically modified histone peptide ligands has become the standard in the field for identifying the preferred ligands, they do not fully represent the biological context of the natural ligands, where the N-terminal histone tails are found protruding from the nucleosome. Furthermore, despite our best efforts, we were unable to produce a crystal structure of the bromodomain in association with an acetylated histone ligand, and this structural information would have been useful for deciphering the ligand binding specificities driving the interaction of *Pf*BDP1 with chromatin.

## 4. Conclusion

In this study, we report the 2.0 Å resolution crystal structure of *Pf*BDP1-BRD (apo state), confirming that it has the conserved bromodomain fold and a functional acetyllysine binding pocket. Importantly, at this resolution our structure had continuous electron density for the ZA loop, a crucial component of the acetyllysine binding pocket, which was absent in the initially deposited structures (Suppl. Fig. 1). A comparison of the *Pf*BDP1-BRD structural features with those of the TIF1α-BRD/H3K27ac complex structure (PDB ID: 3O35) reveals that, like other bromodomains, the predicted acetyllysine binding pocket of the *Pf*BDP1-BRD (333–456) contains a conserved asparagine residue (Asn413), a gatekeeper residue valine (Val 419), and the HIF shelf motif (res 354–356), that are crucial for the specific recognition and binding of acetylated lysine post-translational modifications on histones. We conducted a comprehensive structural analysis to examine the similarities and differences between the acetyllysine binding pockets of the *Pf*BDP1-BRD and human bromodomains. Based on a structural alignment, the 61 bromodomains in humans are divided into eight groups [15]. We compared the *Pf*BDP1-BRD structure to structural models of a single member of each family, that were chosen based on highest sequence identity. Our thorough examination of the BRD binding pockets reveals that, in addition to sharing many structural features with human bromodomains, the *Pf*BDP1-BRD has a distinct chemical composition in the acetyllysine binding pocket. This information may be used to develop a targeted drug inhibitor for *Pf*BDP1-BRD. *Pf*BDP1-BRD was previously demonstrated to bind with histone H3 acetylated at lysine 9 and 14 (H3K9ac and H3K14ac), and this activity was necessary for *Plasmodium falciparum* invasion-related gene activation [24]. However, only a small number of unmodified, acetylated, and methylated lysine residues on histone H3 were used to characterize the unique binding interactions of the *Pf*BDP1-BRD with PTM histone ligands. In addition to the previously reported interactions with acetylated histone H3, the solution NMR titration and ITC binding experiments with acetylated histone ligands presented in this study, show that *Pf*BDP1-BRD preferentially selects for tetra-acetylated histone H4. We also identified weaker associations with multi-acetylated H2A.Z. Our results suggest that *Pf*BDP1 may play other functions in the *P. falciparum* life cycle, and the unique characteristics of its bromodomain binding pocket may be leveraged for the design novel therapeutic compounds to overcome *P. falciparum’s* increasing resistance to presently available medications.

## 5. Materials and methods

### 5.1. Plasmid construction

The DNA sequence encoding *Pf*BDP1-BRD (333–456) region was amplified using polymerase chain reaction (PCR) taking *Pf*BDP1 full length as a template. The PCR amplified *Pf*BDP1-BRD (333–456) region was subsequently cloned into the pDEST15 vector containing the N-terminal glutathione S-transferase (GST) tag using the Gateway cloning approach. The codon-optimized *Pf*BDP1-BRD (303–488) construct cloned in a pGEX-6P-1 vector with an N terminal GST-tag followed by a precision protease site was obtained from GenScript.

### 5.2. Protein overexpression and chromatographic purification

The *Pf*BDP1-BRD (333–456) and *Pf*BDP1-BRD (303–488) constructs in a pGEX-6P-1 vector were transformed into *E. coli* BL21 (DE3) pLysS cells and allowed to grow in a TB medium containing 100 μg/mL ampicillin and 25 μg/mL chloramphenicol at 37°C at OD_600_ 0.8. The cells were induced with 0.2 mM isopropyl β-D-thiogalactopyranoside (IPTG) and induction was allowed to proceed for 16 h at 20°C. The cells were harvested by centrifugation at 5000 RPM for 10 min. The cell pellet was homogenized in a lysis buffer containing 50 mM HEPES (pH 7.5), 500 mM NaCl, 1 mM EDTA, 2 mM DTT, 10% Glycerol, 0.1 mg/mL lysozyme, and a protease inhibitor tablet (Thermo Scientific). The homogenized cells were lysed by sonication and centrifuged at a speed of 11,000 RPM for 60 min at 4°C to pellet the insoluble material. The obtained supernatant was incubated at 4°C for 2 h with 10 mL of Glutathione Beads packed in a 25 mL chromatography column (G Biosciences). After incubation beads were washed with 200 mL wash buffer containing 20 mM HEPES (pH 7.5), 500 mM NaCl, 1 mM EDTA, and 2 mM DTT followed by GST-tag cleavage using Precision protease at 4°C for 14 h. After GST cleavage, the *Pf*BD1-BRD proteins were eluted with wash buffer. SDS-PAGE gels stained with GelCode Blue (Thermo Scientific) were used to confirm the purity of the *Pf*BDP1-BRD protein samples. The protein concentration was determined by absorbance at 280 nm using a NanoDrop (Thermo Scientific) and an extinction coefficient of 18,910 M^−1^ cm^−1^ for *Pf*BDP1-BRD (333–456) and 29,910 M^−1^ cm^−1^ for *Pf*BDP1-BRD (303–488). For X-ray crystallization experiments the protein was further purified by size-exclusion chromatography (SEC) using a HiPrep 16/60 Sephacryl S-100 HR column at 4°C in a buffer containing 20 mM HEPES (pH 7.5), 150 mM NaCl, and 2 mM DTT.

### 5.3. Histone peptide synthesis

All the unmodified and acetylated histone peptides were purchased from GenScript (Piscataway, NJ, USA). The synthesized peptides were purified by HPLC to achieve 98% purity and present in lyophilized form with TFA salt. The identity of each peptide was confirmed with mass spectrometry and the sequence of the peptides with modifications are listed in Table 3. Despite multiple attempts the *P. falciparum* H2A. ZK11acK15acK19ac could not be synthesized.

### 5.4. Isothermal titration calorimetry

Isothermal titration calorimetry (ITC) experiments were carried out using a MicroCal PEAQ-ITC and a MicroCal iTC200 (Malvern Panalytical). The purified *Pf*BDP1-BRD proteins (containing residues 303–488 or 333–456) were dialyzed for 48 h at 4°C into ITC buffer containing 20 mM sodium phosphate buffer pH 7.5, 150 mM NaCl, and 1 mM TCEP. The dialyzed *Pf*BDP1-BRD (303–488 or 333–456) proteins were added to the sample cell at concentrations of 100–200 μM, and between 2.5 and 5.0 mM of each histone peptide in the ITC dialysis buffer was placed in the injection syringe. A total of 20 injections were carried out, with the parameters set as follows. The first injection had a volume of 0.5 μL, a reaction time of 0.4 s, followed by a 180s delay. The remaining 19 injections were completed with an injection volume of 2 μL, a reaction time of 4 s, and a 150 s spacing time between each injection. All ITC runs were carried out at 5°C with a differential power (DP) value of 10, the feedback set to high, at a stirring speed of 750 RPM, and with an initial delay for the reaction set to 60 s.

The ITC data obtained were analyzed using the MicroCal PEAQ-ITC 200 analysis software provided with the instrument (Malvern Panalytical). The heat of dilutions for the buffer and peptide ligands were obtained from titrating buffer into buffer, buffer into *Pf*BDP1-BRD protein (303–488 and 333–456), and histone peptide into buffer. The heats of dilution for the histone peptides were subtracted from the raw isotherms obtained from the histone peptide and *Pf*BDP1-BRD (303–488 and 333–456) protein titrations. The first injection was discarded from the data analysis, and the data was integrated using a 1:1 binding model assuming a single set of identical binding sites. A Marquandt nonlinear least-squares analysis was used to calculate the binding affinities and N values from the normalized heat changes. Binding experiments were repeated in triplicate for each ligand and the mean K_D_ was calculated from the average of the three runs, with the standard deviation coming from the mean. Non-binding ligand experiments were repeated in duplicate.

### 5.5. Nuclear magnetic resonance spectroscopy

Chemical shift perturbation experiments were performed using 0.3–0.5 mM of uniformly ^15^N-labeled *Pf*BDP1-BRD (303–488) in NMR buffer containing 20 mM Tris-HCl pH 6.8, 150 mM NaCl, 10 mM DTT and 10% D_2_O. Titration mixtures of the *Pf*BDP1-BRD (303–488) and each of the modified histone peptides (res 1–24) unmodified H3, H3K9ac, H3K14ac, H3K9acK14ac, unmodified H4, H4K5ac, H4K12ac, H4K5acK8acK12acK16ac, unmodified H2A.Z, H2A.ZK11ac, unmodified H2B.Z, H2B.ZK3acK8acK13acK14acK18ac were prepared at molar ratios of 1:0, 1:0.5, 1:1, 1:2.5, and 1:5 M, in a total volume of 80 μL, The sample mixtures were then transferred into 1.7 mm NMR tubes (Bruker). Two-dimensional ^15^N−^1^H HSQC (heteronuclear single quantum coherence) experiments for all samples were acquired on Bruker Avance III spectrometer equipped with a cryogenic 1.7 mm probe. The temperature of the samples was regulated at 25°C throughout data collection. All spectra were processed using TopSpin (Bruker) as described previously [43] and analyzed using NMRFAM-SPARKY [48].

### 5.6. Crystallization of PfBDP1-BRD (333–456)

For crystallization, the *Pf*BDP1-BRD (333–456) was concentrated to ~15 mg/mL in a buffer containing 20 mM HEPES (pH 7.5), 150 mM NaCl and 2 mM DTT. The crystallization trials were done using commercially available conditions such as Index HT Screen (Hampton Research), Crystal Screen HT (Hampton Research), JSCG-plus HT-96 (Molecular Dimension), and the Ligand Friendly Screen HT-96 (Molecular Dimension). Crystals were set up in a 96-well sitting drop plate (96-well Swissci 3-well midi UVXPO crystallization plate, HR3–125 Hampton research) with a reservoir volume of 40 μL and a drop volume of 2 μL. The *Pf*BDP1-BRD (333–456) protein at 10 mg/mL with 5.3 mM of the histone H3K14ac (res 1–19) ligand was mixed in ratios of either 1:1 or 1:3 with the mother liquor and grown using a sitting drop vapor diffusion method at 4°C.

Initial *Pf*BDP1-BRD (333–456) protein crystals were obtained after three days of incubation at 4°C in condition D7 from the JCSG-plus HT-96 crystal screen 0.2 M Lithium sulfate, 0.1 M Tris pH 8.5, 40% v/v PEG400, 100 μL 1,8-ANS. The crystal conditions were optimized in a 24-well VDX hanging drop tray (Hampton Research) at 4°C. The final crystals grew in a 2 μL drop containing 1 μL of 12 mg/mL *Pf*BDP1-BRD mixed with 5.3 mM of histone H3K14ac (res 1–19) ligand, and 1 μL of mother liquor containing 0.3 M lithium sulfate, 0.1 M Tris-HCL pH 8.5, and 38% PEG400. The crystal was harvested in a 75 μm Dual Thickness Microloop LD with an 18 mm pin inserted into a B1A (ALS style) reusable goniometer base from MiTeGen (Ithaca, NY, USA). The crystals were flash frozen in liquid nitrogen at 100 K. A high-resolution dataset was collected at the Advanced Light Source (ALS) beamline 4.2.2 and recorded on an RDI CMOS-8M detector. The data were processed using XDS [44], and an initial structure solution was obtained by molecular replacement using PHASER with PDBID: 5ULC as the search model [45]. Iterative rounds of model building, and refinement were carried out with PHENIX and COOT [46], and the final model of the *Pf*BDP1-BRD was deposited into the PDB under PDBID: 7M97. The structure figures and structural superpositions were prepared using PyMOL [47].

## Supplementary Material

Supplementary data

## Figures and Tables

**Table 1:** Data collection and structure refinement statistics for the *Pf*BDP1-BRD.

Data collection (PDB ID: 7M97)	
No. of crystals	1
Beamline	ALS 4.2.2
Wavelength	0.9762
Detector	RDI CMOS_8M
Crystal to detector distance (mm)	225
Rotation range per image	0.2
Total rotation range (°)	180
Exposure time per image (s)	0.5
Resolution range (Å)	34.97–2.0 (2.072–2.000)
Space group	I 2 2 2
Unit cell parameters	
a,b,c (Å)	38.051, 88.667, 106.797
α, β, γ (°)	90, 90, 90
Unique reflections	12,597 (1203)
R_work_/R_free_ (%)	19.49/23.65
Multiplicity	7.0 (6.4)
Mean I/s (I)	19.24 (2.93)
Completeness (%)	99.63 (99.25)
R_merge_ (%)	2.602 (26.72)
Wilson B factor (Å)	29.36
Number of non-hydrogen atoms	
Macromolecules	1006
Solvent	110
Protein residues	124
RMSD	
Bond length (Å)	0.008
Bond angles (°)	0.89
Ramachandran plot	
Favored regions (%)	98.36
Allowed regions (%)	1.64
Outliers (%)	0.00

**Table 2:** Structural analysis of the *Pf*BDP1-BRD (PDB ID: 7M97) compared to human bromodomains in families I–VIII.

Human bromodomain protein	PDB ID	Sequence identity %	RMSD value (calculated using PDBeFold)	Number of hydrophobic residues in binding pocket	Number of acidic residues in binding pocket	Number of basic residues in binding pocket
Family V TIF1α	3O33	39.58	0.88 Å	18	04	0
Family I CECR2	3NXB	36.76	0.92 Å	06	02	04
Family II BRD4	2OUO	36.59	1.21 Å	20	02	02
Family III CREBBP	3DWY	48.44	1.33 Å	10	02	01
Family VIII PB1(3)	3K2J	41.07	1.48 Å	14	04	03
Family IV ATAD2B	3LXJ	33.75	1.78 Å	15	03	03
Family VII TAF1	3UV5	38.10	2.00 Å	14	04	01
Family VI TRIM28	2RO1	22.95	2.07 Å	08	05	03

**Table 3:** Binding affinities of the *Pf*BDP1-BRD with acetylated histone peptides measured by ITC.

Histone peptide (1–24)	Sequence	*Pf*BDP1-BRD K_D_ (μM)	N-value
Histone H3 peptides			
Mono-acetylated histone H3 peptides			
H3 unmodified	ARTKQTARKSTGGKAPRKQLATKA	No Binding	–
H3K9ac	ARTKQTAR(*Kac*)STGGKAPRKQLATKA	No Binding	–
H3K14ac	ARTKQTARKSTGG(*Kac*)APRKQLATKA	1215.00 ± 162.3	1.0
Di-acetylated histone H3 peptides			
H3K9acK14ac	ARTKQTAR(*Kac*)STGG(*K*ac)APRKQLATKA	1125 ± 181	1.0
Histone H4 peptides			
Mono-acetylated histone H4 peptides			
H4 unmodified	SGRGKGGKGLGKGGAKRHRKVLRD	No Binding	–
H4K5ac	SGRG(*Kac*)GGKGLGKGGAKRHRKVLRD	1210 ± 208	1.0
H4K8ac	SGRGKGG(*Kac*)GLGKGGAKRHRKVLRD	No Binding	–
H4K12ac	SGRGKGGKGLG(*Kac*)GGAKRHRKVLRD	1070 ± 158	1.0
H4K16ac	SGRGKGGKGLGKGGA(*Kac*)RHRKVLRD	No Binding	–
H4K20ac	SGRGKGGKGLGKGGAKRHR(*Kac*)VLRD	1130 ± 144	1.0
Diacetylated histone H4 peptides			
H4K5acK8ac	SGRG(*Kac*)GG(*Kac*)GLGKGGAKRHRKVLRD	1510 ± 168	1.0
H4K5acK12ac	SGRG(*Kac*)GGKGLG(*Kac*)GGAKRHRKVLRD	621.0 ± 28.3	1.0
Tetra acetylated histone H4 peptides			
H4K5acK8acK12acK16ac	SGRG(*Kac*)GG(*Kac*)GLG(*Kac*)GGA(*Kac*)RHR(*Kac*)VLRD	173.0 ± 11.3	1.0
Histone H2A.Z peptides			
Mono-acetylated histone H2A.Z peptides			
H2A.Z unmodified	MEVPGVIGGKVGGKVGGKVLGLG	No Binding	–
H2A.ZK11ac	MEVPGVIGG(*Kac*)VGGKVGGKVLGLG	No Binding	–
H2A.ZK15ac	MEVPGVIGGKVGG(*Kac*)VGGKVLGLG	No Binding	–
H2A.ZK19ac	MEVPGVIGGKVGGKVGG(*Kac*)VLGLG	No Binding	–
Histone H2B.Z peptides			
Mono-acetylated histone H2B.Z peptides			
H2B.Z unmodified	SGKGPAQKSQAAKKTAGKTLGPR	No Binding	–
H2B.ZK3ac	SG(*Kac*)GPAQKSQAAKKTAGKTLGPR	No Binding	–
H2B.ZK8ac	SGKGPAQ(*Kac*)SQAAKKTAGKTLGPR	No Binding	–
H2B.ZK13ac	SGKGPAQKSQAA(*Kac*)KTAGKTLGPR	No Binding	–
H2B.ZK14ac	SGKGPAQKSQAAK(*Kac*)TAGKTLGPR	No Binding	–
H2B.ZK18ac	SGKGPAQKSQAAKKTAG(*Kac*)TLGPR	No Binding	–
Penta-acetylated histone H2B.Z peptides			
H2B.ZK3acK8acK13acK14acK18ac	SG(*Kac*)GPAQ(*Kac*)SQAA(*Kac*)(*Kac*)TAG(*Kac*)TLGPR	1303 ± 111.3	1.0
