# Supplementary material for: Structural insights into acetylated histone ligand recognition by the BDP1 bromodomain of Plasmodium falciparum
Source: Int J Biol Macromol. Author manuscript; Available in PMC 2023 Apr 12. (PMC10093686; doi:10.1016/j.ijbiomac.2022.10.247)
Supplement: Supplementary data [file NIHMS1887615-supplement-Supplementary_data.docx]

**Structural insights into acetylated histone ligand recognition by the BDP1 bromodomain of *Plasmodium falciparum***

Ajit Kumar Singh^1^, Margaret Phillips^1^, Saleh Alkrimi^2^, Marco Tonelli^3^, Samuel P. Boyson^2^, Kiera L. Malone^1^, Jay C. Nix^4^, and Karen C. Glass*^1,2^

**Supporting Information**

^1^Department of Pharmacology, Larner College of Medicine, University of Vermont, Burlington, VT, 05405, USA

^2^Department of Pharmaceutical Sciences, Albany College of Pharmacy and Health Sciences, Colchester, VT, 05446, USA

^3^NMRFAM and Department of Biochemistry, University of Wisconsin-Madison, Madison, Wisconsin, 53706, USA

^4^Molecular Biology Consortium, Advanced Light Source, Berkeley, CA, 94720, USA

*Corresponding author

**Contents**

Suppl. Fig. 1. Crystal structure of *Pf*BDP1-BRD at 2.0 Å. 3

Suppl. Fig. 2A. Sequence alignment of *Pf*BDP1-BRD with human bromodomain. 4

Suppl. Fig. 2B. Structural alignment of *Pf*BDP1-BRD with human bromodomain. 5

Suppl. Fig. 3. ITC enthalpy plots for the binding of *Pf*BDP1-BRD with acetylated histone peptides. 6

Suppl. Fig. 4. Interaction of *Pf*BDP1-BRD with unmodified histone peptide 7

References 8

**
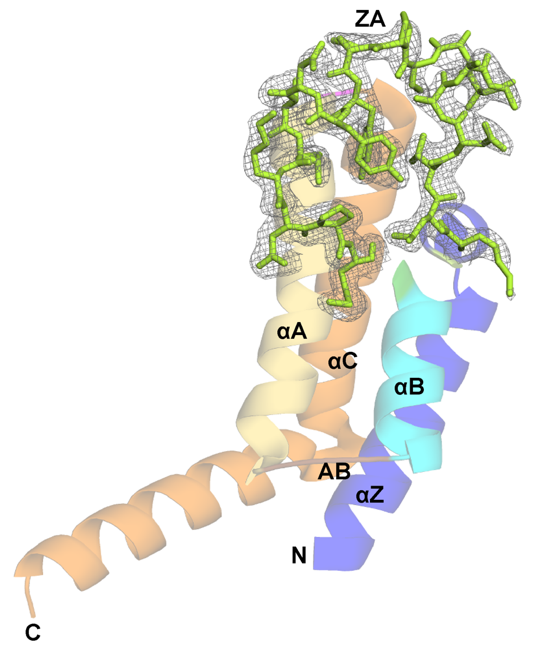
**

Suppl. Fig. 1. Crystal structure of *Pf*BDP1-BRD at 2.0 Å. The 2F_O_ - F_C_ electron density map for ZA loop in *Pf*BDP1-BRD is displayed at 1.0σ contour level.


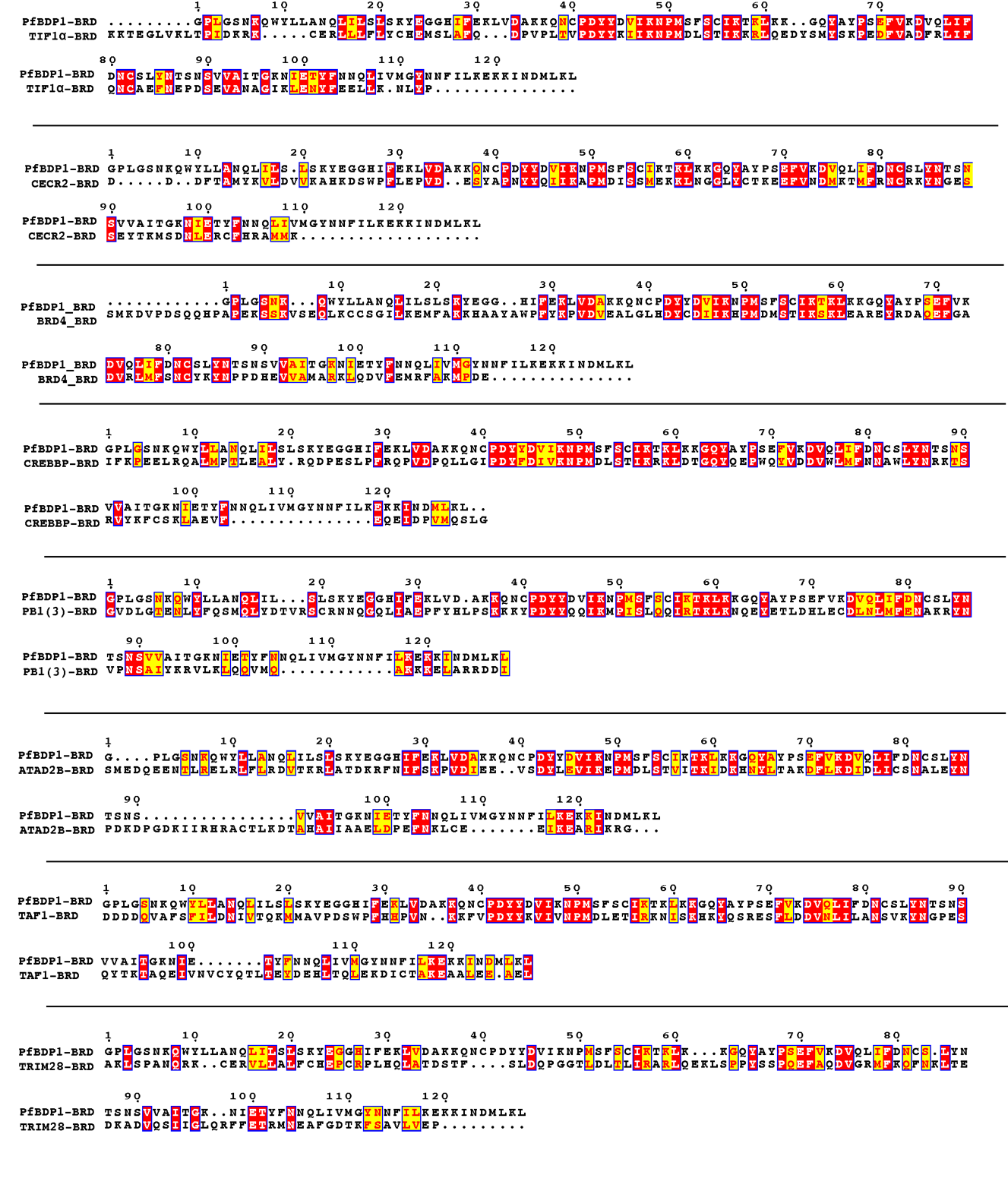


Suppl. Fig. 2A. Sequence alignment of *Pf*BDP1-BRD with human bromodomain. The sequence alignment of the *Pf*BDP1-BRD with the human bromodomains if TIF1α (PDB ID: 3033), CECR2 (PDB ID: 3NXB), BRD4 (PDB ID: 2OUO), CREBBP (PDB ID: 3DWY), PB1(3) (PDB ID: 3K2J), ATAD2B (PDB ID: 3LXJ), TAF1 (PDB ID: 3UV5), and TAF1 (PDB ID: 2RO1) was done using CLUSTAL W (version 1.83) multiple sequence alignment [1]. Sequence alignment figures were made using ESPript 3.0 [2]


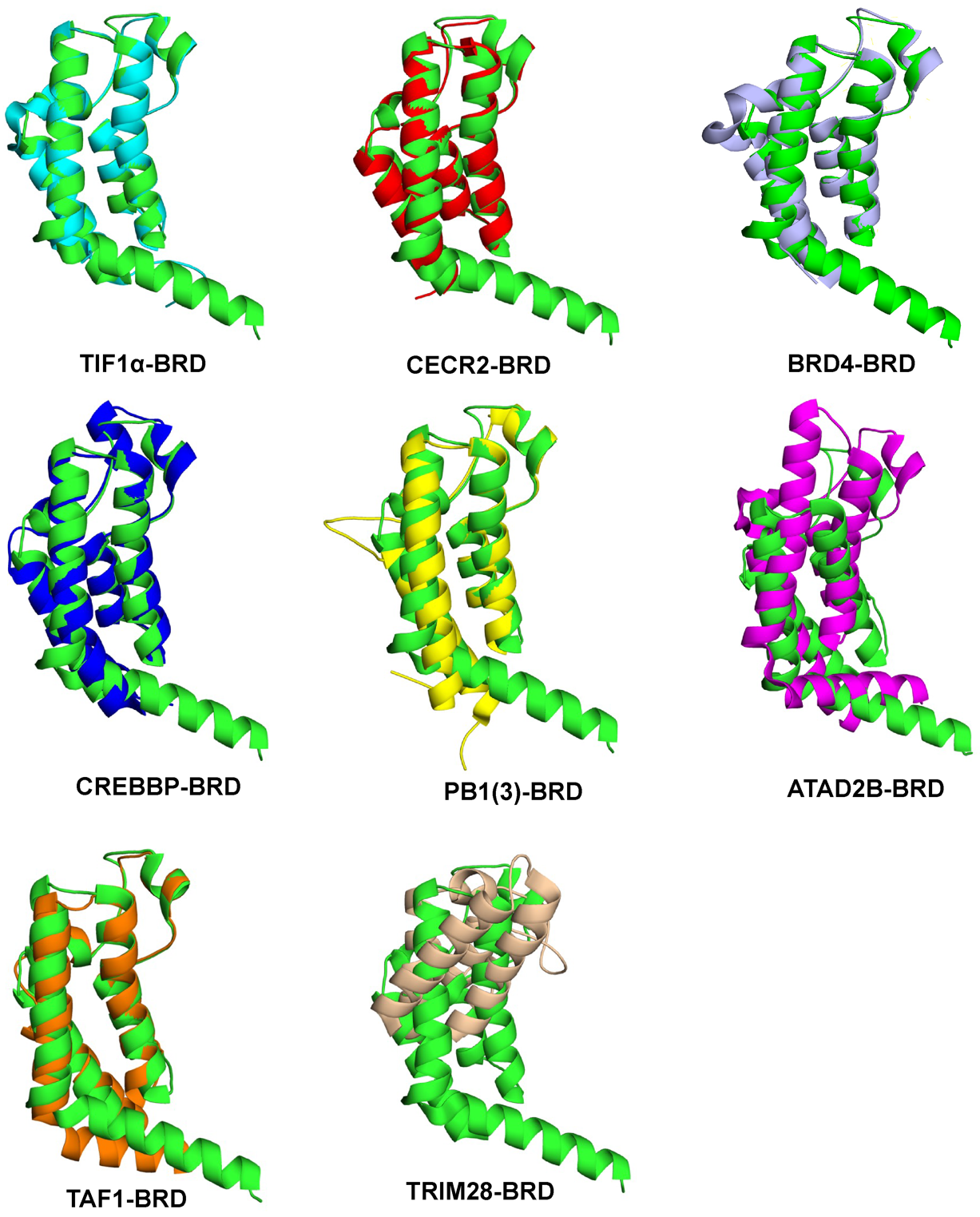


Suppl. Fig. 2B. Structural alignment of *Pf*BDP1-BRD with human bromodomains. The structural alignment of the *Pf*BDP1-BRD (green) with the human bromodomains of TIF1α (PDB ID: 3033 cyan), CECR2 (PDB ID: 3NXB red), BRD4 (PDB ID: 2OUO), CREBBP (PDB ID: 3DWY blue), PB1(3) (PDB ID: 3K2J yellow), ATAD2B (PDB ID: 3LXJ magenta), TAF1 (PDB ID: 3UV5 orange), and TAF1 (PDB ID: 2RO1 brown) was done using PyMOL [3] and the RMSD was calculated using the PDBeFold online server [4]


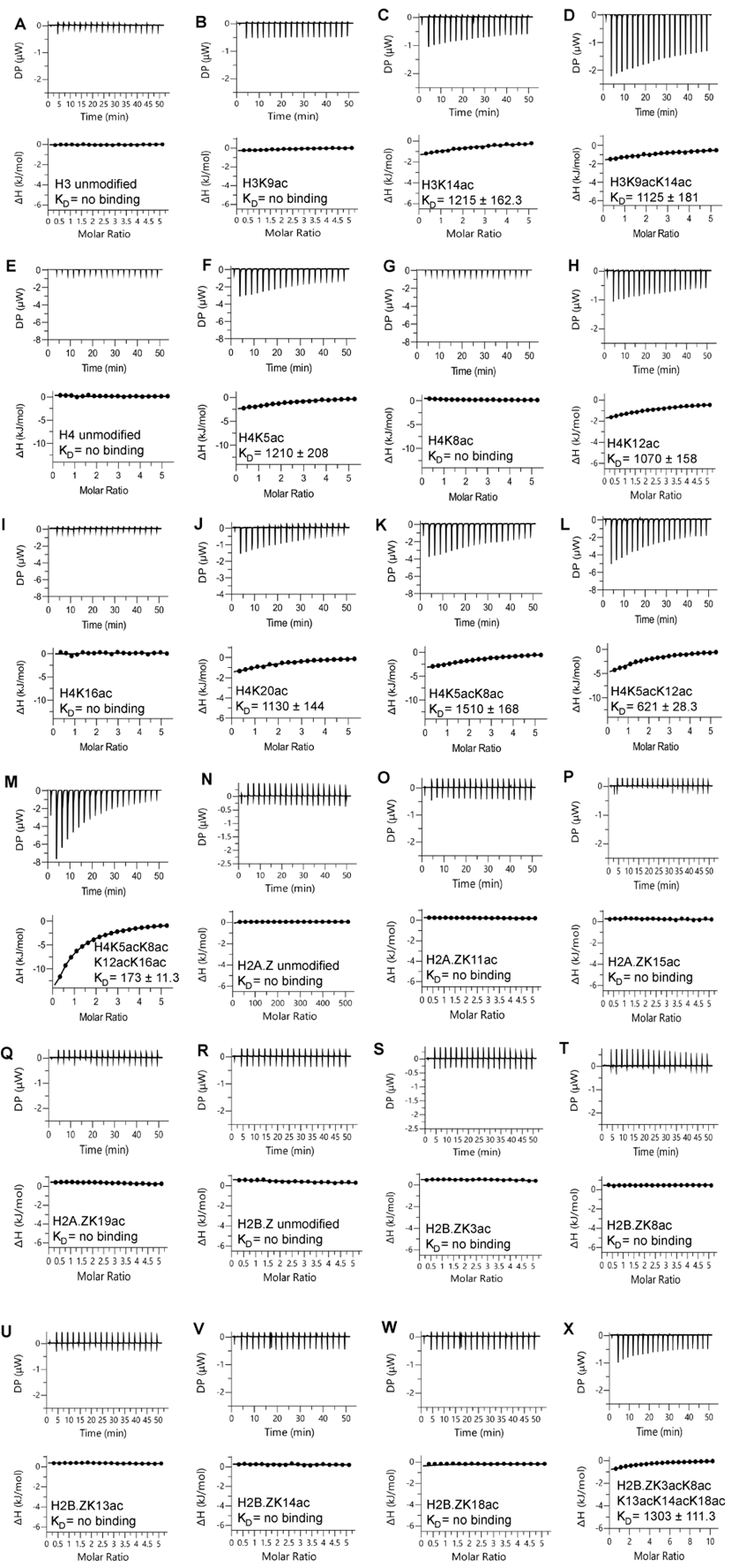


Suppl. Fig. 3. ITC enthalpy plots for the binding of *Pf*BDP1-BRD with acetylated histone peptides. (A-D) *Pf*BDP1-BRD with acetylated histone H3 peptides. (E-M) *Pf*BDP1-BRD with acetylated histone H4 peptides. (N-Q) *Pf*BDP1-BRD with acetylated histone H2A.Z peptides. (R-X) *Pf*BDP1-BRD with acetylated histone H2B.Z peptides


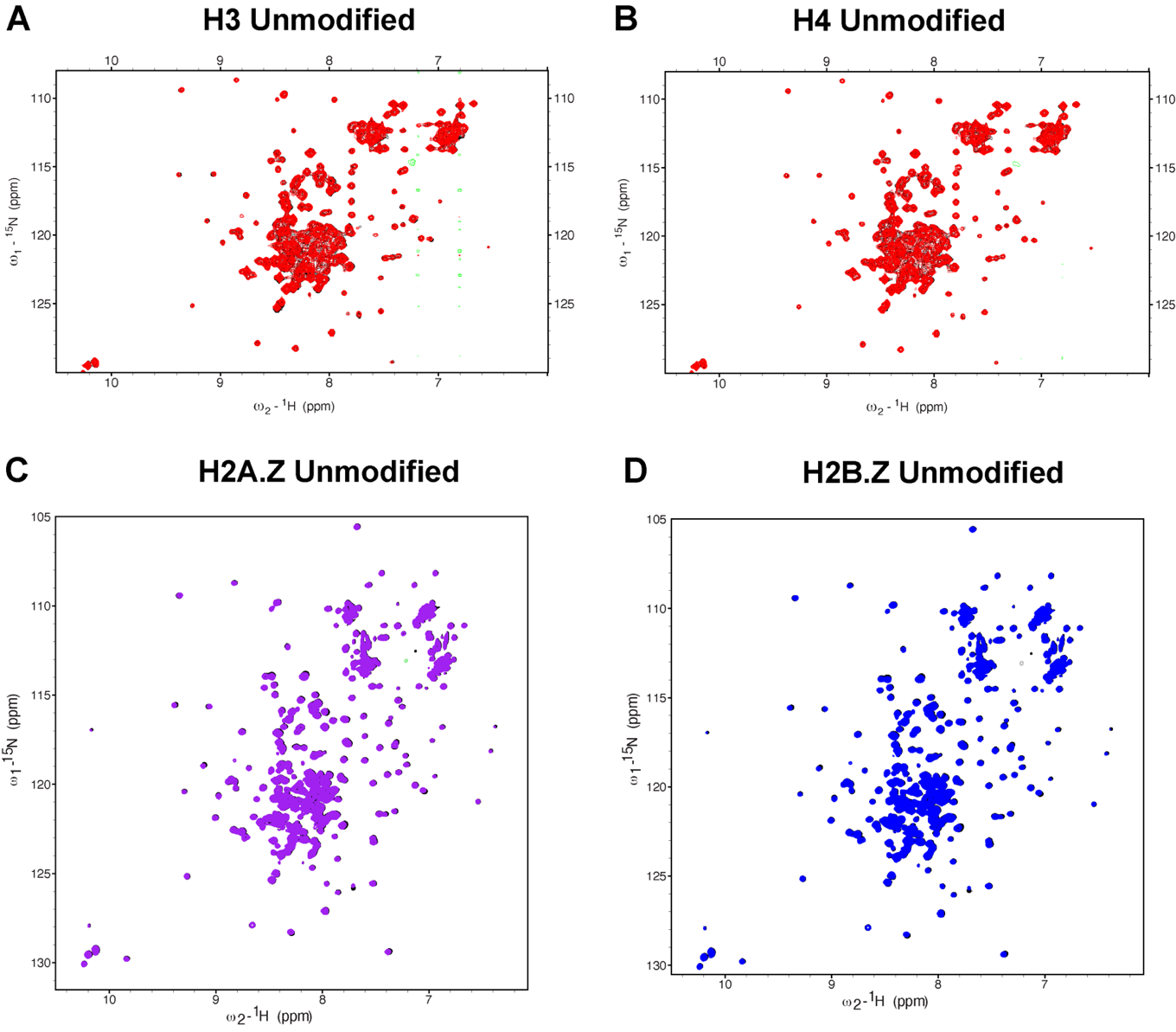


Suppl. Fig. 4. Interaction of the *Pf*BDP1-BRD with unmodified histone peptides**.** Superimposed 2D ^15^N-^1^H HSQC spectra of the ^15^N-labeled *Pf*BDP1-BRD collected in titration experiments with the indicated histone peptides. The peaks of the *Pf*BDP1-BRD apo protein are shown in black. The titration of *Pf*BDP1-BRD with histone peptides was performed at 1:0 molar ratio (black), and in 1:5 molar ratio of unmodified histone H3 and H4 (red A-B), Unmodified H2A.Z (purple C), and unmodified H2B.Z (blue D). Each spectrum is labeled with the histone peptide used for the titration.

# References

[1] R. Chenna, H. Sugawara, T. Koike, R. Lopez, T.J. Gibson, D.G. Higgins, J.D. Thompson, Multiple sequence alignment with the Clustal series of programs, Nucleic Acids Res 31(13) (2003) 3497-500.

[2] X. Robert, P. Gouet, Deciphering key features in protein structures with the new ENDscript server, Nucleic Acids Res 42(Web Server issue) (2014) W320-4.

[3] W.L. DeLano, The PyMOL Molecular Graphics System in, DeLano Scientific, Palo Alto, CA (2002).

[4] E.K.a.K. Henrick, Protein structure comparison service PDBeFold at European Bioinformatics Institute.
